# Prognostic value of stress perfusion cardiac magnetic resonance in patients with prediabetes and suspected coronary artery disease

**DOI:** 10.1371/journal.pone.0311875

**Published:** 2024-10-10

**Authors:** Norapat Leungratanamart, Kasinee Wanitchung, Sumet Prechawuttidech, Khemajira Karaketklang, Yodying Kaolawanich

**Affiliations:** 1 Division of Cardiology, Department of Medicine, Faculty of Medicine Siriraj Hospital, Mahidol University, Bangkok, Thailand; 2 Department of Medicine, Faculty of Medicine Siriraj Hospital, Mahidol University, Bangkok, Thailand; Osaka University Graduate School of Medicine, JAPAN

## Abstract

**Background:**

Stress perfusion cardiac magnetic resonance (CMR) is an accurate and comprehensive modality for evaluating patients with suspected coronary artery disease (CAD), but its prognostic value in prediabetic patients is uncertain.

**Methods:**

This retrospective study included 452 consecutive prediabetic patients without prior diagnoses of CAD who underwent adenosine stress perfusion CMR. The primary endpoint was major adverse cardiovascular events (MACE), defined as cardiovascular death, nonfatal myocardial infarction (MI), hospitalization for heart failure, ischemic stroke, and late coronary revascularization (>90 days post-CMR). The secondary endpoint was a composite of cardiovascular death, nonfatal MI, and hospitalization for heart failure.

**Results:**

The mean age was 68±11 years (49% male). Over a median follow-up time of 8.1 (IQR 5.7, 10.4) years, 55 patients experienced MACE, and 24 met the secondary endpoint. Patients with inducible ischemia had significantly greater annualized event rates for MACE (5.7% vs. 0.7%, *p*<0.001) and for the secondary endpoint (2.0% vs. 0.3%, *p*<0.001) than those without ischemia. Multivariable analysis revealed inducible ischemia as a consistent predictor for MACE (HR 3.36, 95%CI 1.90–5.94, *p*<0.001) and for the secondary endpoint (HR 2.89, 95%CI 1.22–6.80, *p* = 0.01). Late gadolinium enhancement (LGE) was an independent predictor of the secondary endpoint (HR 3.56, 95%CI 1.25–10.13; *p* = 0.02). Incorporating inducible ischemia and LGE data significantly improved the model’s ability to discriminate MACE risk (C-statistic increase from 0.77 to 0.83; net reclassification improvement 0.42; integrated discrimination improvement 0.05).

**Conclusion:**

Stress perfusion CMR offers substantial independent prognostic value and effectively aids in reclassifying cardiovascular risk among prediabetic patients with suspected CAD.

## Introduction

Prediabetes denotes a medical condition marked by elevated levels of blood glucose that fall below the diagnostic threshold for diabetes, yet pose an increased susceptibility to the development of diabetes mellitus. This category pertains to individuals exhibiting impaired fasting glucose (IFG), impaired glucose tolerance (IGT), and/or HbA1c levels within the range of 5.7 to 6.4% [1]. As of 2021, the global prevalence of impaired fasting glucose stood at 5.8%, equating to 298 million individuals, with projections indicating an increase to 6.5% (414 million) by 2045 [2].

Individuals with prediabetes face an increased risk of cardiovascular diseases, particularly coronary artery disease (CAD) [3]. This demographic group also has a higher risk of cardiovascular mortality [4, 5], emphasizing the need for accurate diagnosis and risk stratification of CAD in prediabetic patients.

The utilization of stress perfusion cardiac magnetic resonance (CMR) is on the rise within clinical practice, particularly among patients who are either diagnosed with or suspected to have CAD [6–8]. Its established diagnostic accuracy underscores its importance [9]. Stress perfusion CMR offers the distinct advantage of not only assessing inducible myocardial ischemia but also evaluating cardiac function and delineating the presence and extent of myocardial scarring using the late gadolinium enhancement (LGE) technique, all without radiation exposure. The evaluation of ischemia using this method is unaffected by balanced ischemia or microvascular dysfunction, making it the preferred choice for assessing myocardial fibrosis and identifying previous ischemic damage [10]. Although the adenosine stress perfusion CMR has been shown to have prognostic value in diabetic patients with known or suspected CAD [11, 12], its prognostic relevance in the prediabetic population is underexplored.

This study aims to evaluate the prognostic value of adenosine stress perfusion CMR in patients with prediabetes and suspected CAD, as well as to assess its incremental risk stratification over conventional risk factors.

## Methods

### Study population

This retrospective study included consecutive patients aged 18 years or older with prediabetes and without known CAD, who were referred for adenosine stress perfusion CMR between February 2011 and December 2013 at our outpatient center. Prediabetes was defined in accordance with the American Diabetes Association as any or all of the following: fasting plasma glucose (FPG) 100–125 mg/dL; HbA1c 5.7–6.4%; 2-hour plasma glucose during 75-g OGTT 140–199 mg/dL [1].

The exclusion criteria were as follows: (i) a history of CAD, including percutaneous coronary intervention, coronary artery bypass graft, or myocardial infarction (MI). MI was confirmed either through documentation in medical records or by the presence of significant Q waves on a 12-lead electrocardiogram within a coronary territory; (ii) known cardiomyopathy, congenital heart disease, or severe primary valvular disease; (iii) incomplete CMR examination. Patients were considered lost to follow-up if they had not experienced any component of the primary or secondary endpoints and had less than 6 months of follow-up.

Clinical data were collected through medical records and examination on the day of the CMR. The history of cardiovascular comorbidities, such as hypertension and hyperlipidemia, was defined by current guidelines [13–15]. This study was approved by the Siriraj Institutional Review Board (SIRB) (COA no. 138/2022). Written informed consent to participate was not obtained from study patients due to the retrospective and confidentiality-preserving design of this study. Data were accessed for research purposes between April 1^st^ and August 1^st^, 2022. The study adhered to the ethical principles of the 1964 Declaration of Helsinki.

### CMR protocol

The CMR studies were performed with a 1.5 Tesla Philips Achieva XR scanner (Philips Medical Systems, Best, The Netherlands) to evaluate cardiac function, myocardial perfusion, and LGE.

The images for the cardiac functional study were acquired using the steady-state free precession (SSFP) technique in multiple slice short-axis views, 2-chamber, 3-chamber, and 4-chamber views. The parameters for cardiac function study were as follows: echo time (TE) of 1.8 milliseconds (ms), repetitive time (TR) of 3.7 ms, number of excitations of 2, field of view (FOV) measuring 390 x 312 mm, matrix size of 256 x 240, reconstruction pixels measuring 1.52 x 1.21, slice thickness of 8 mm, and a flip angle of 70 degrees.

The myocardial first-pass perfusion study involved a 4-minute infusion of adenosine at 140 μg/kg/min, followed by the injection of 0.05 mmol/kg of gadolinium contrast agent (Magnevist, Bayer Schering Pharma, Berlin, Germany) and a saline bolus, administered at a rate of 4 mL/s [16]. Using an ECG-triggered, SSFP, inversion-recovery, single-shot, turbo gradient-echo sequence, three short-axis slices at basal, mid, and apical left ventricular (LV) levels were obtained. Image parameters included a TE of 1.32 ms, TR of 2.6 ms, flip angle of 50 degrees, slice thickness of 8 mm, FOV of 270 mm, and reconstructed FOV of 320 mm.

LGE images were acquired 10 minutes after administering a second dose of gadolinium (0.1 mmol/kg, at a rate of 4 mL/s) using the 3D segmented-gradient-echo inversion-recovery sequence. These images were obtained in multiple short-axis slices, as well as in long-axis, 2-chamber, and 4-chamber views, similar to the functional images. Image parameters included a TE of 1.25 ms, TR of 4.1 ms, a flip angle of 15 degrees, FOV of 303 x 384 mm, matrix size of 240 x 256, in-plane resolution of 1.26 x 1.5 mm, slice thickness of 8 mm, and a sensitivity-encoding factor of 1.5.

### Image analysis

Quantitative measurements of LV volume, ejection fraction (EF), and mass were obtained from the stack of short-axis SSFP cine images [17]. Perfusion and LGE images were evaluated using visual assessment and consensus by CMR-trained physicians who were unaware of the clinical and follow-up information. Perfusion images were examined, with visualization of each of the 16 segments (excluding segment-17 at the apex). Inducible ischemia was characterized as a subendocardial perfusion defect meeting specific criteria: persistence beyond peak myocardial enhancement and for several RR intervals, width exceeding two pixels, following one or more coronary arteries, and absence of LGE in the corresponding segment [17]. LGE was deemed to be present only when it was confirmed in both the short-axis and in at least one other orthogonal plane [17]. The total number of LGE segments was determined utilizing the American Heart Association 17-segment model [18].

### Clinical follow-up

Follow-up data were gathered from clinical visits and medical records. Clinical event adjudication was performed with complete blinding to both clinical and CMR data. The primary endpoint comprised major adverse cardiovascular events (MACE), defined as a composite of cardiovascular death, nonfatal MI, hospitalization for heart failure, ischemic stroke, and coronary revascularization. The secondary endpoint comprised a composite of cardiovascular death, nonfatal MI, and hospitalization for heart failure. Cardiovascular death was defined in accordance with established published criteria [19]. In situations where patients encountered multiple events, only the initial event was taken into account for event-free survival analysis. For patients who underwent coronary revascularization within 90 days following the CMR index examination, peri-procedural events (such as MI, heart failure, or death) were not included in the analysis.

### Statistical analysis

Statistical analyses were conducted utilizing IBM SPSS Statistics for Windows, version 20.0 (IBM Corp., Armonk, NY, USA). Continuous variables were expressed as mean ± standard deviation (SD). Categorical variables were presented as absolute numbers and percentages. Differences in clinical baseline and CMR characteristics between patients with and without inducible myocardial ischemia were compared using the student’s unpaired t-test for continuous variables and the chi-square test or Fisher’s exact test for categorical variables, as applicable.

The Kaplan–Meier method was used to estimate cumulative incidence rates of the outcomes between groups and compared with the log-rank test. Univariable predictors from baseline characteristics and CMR parameters were assessed through Cox-regression analysis to analyze the predictors of primary and secondary endpoints. Variables with a p-value <0.05 in the univariable analysis were then entered into the multivariable analysis. To evaluate the prognostic value of inducible ischemia, two multivariable models were developed: Model 1 included inducible ischemia as a categorical variable (presence or absence), while Model 2 included inducible ischemia as a continuous variable (per-segment extent).

The discriminatory ability of each model to predict MACE was assessed using Harrell’s C-statistic both at baseline and after incorporating ischemia and LGE. The incremental predictive contribution of ischemia and LGE was determined through Harrell’s C-statistic increment, categorical net reclassification improvement (NRI), and integrative discrimination index (IDI). The survC1 and survIDINRI packages of R software, version 4.3.2, were employed for the computation of the C-statistic, NRI, and IDI.

The hazard ratios (HRs) and 95% confidence intervals (CIs) were calculated, with a p-value <0.05 considered statistically significant.

## Results

### Patient characteristics

**Fig 1:**
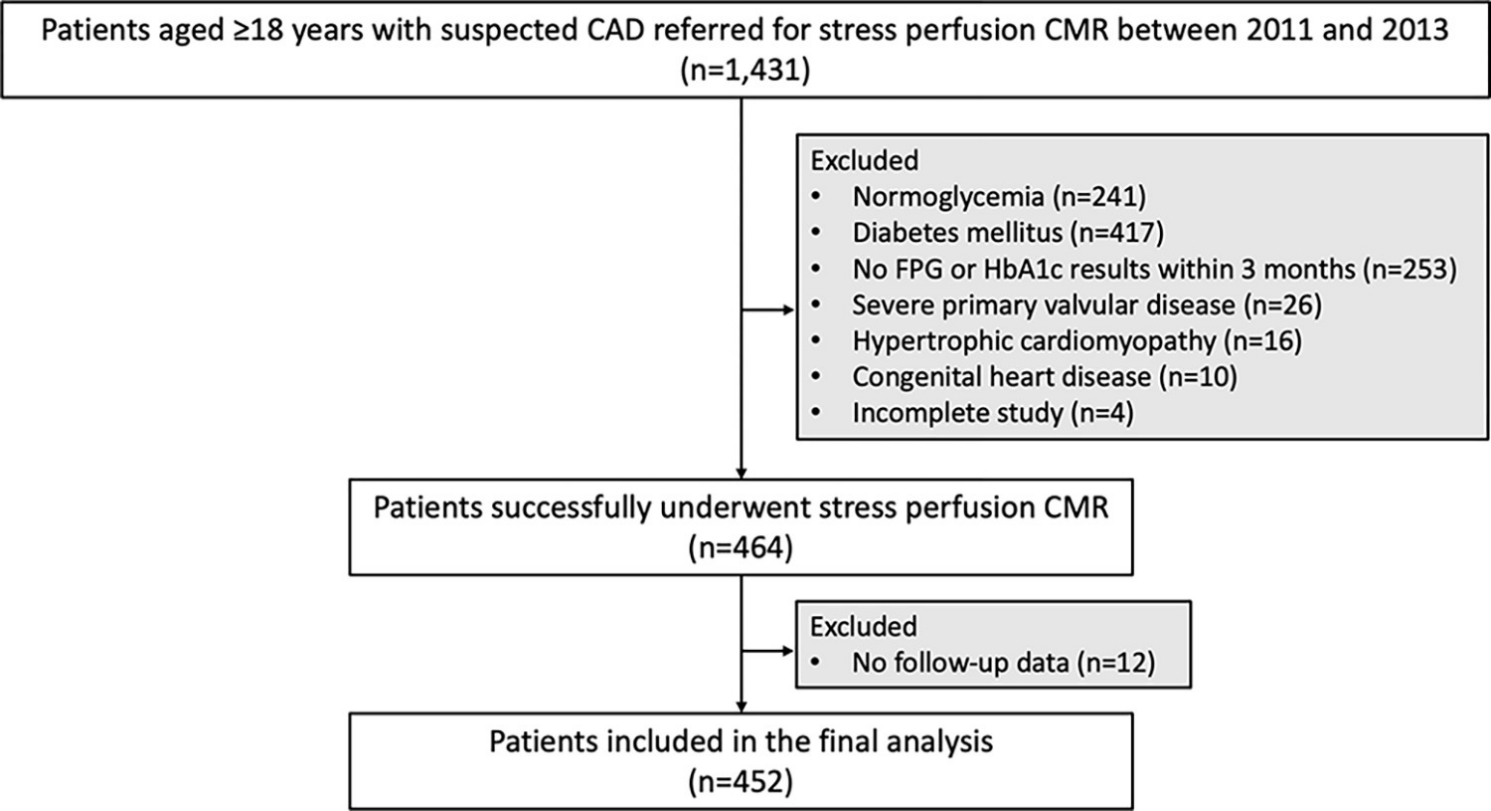
Study flow chart. **Abbreviations:** CAD, coronary artery disease; CMR, cardiac magnetic resonance; FPG, fasting plasma glucose; HbA1c, hemoglobin A1c.

Among the 1,431 patients without known CAD referred for adenosine stress perfusion CMR, 464 were confirmed to have prediabetes using the American Diabetes Association criteria within 3 months of the CMR examination. The flowchart of study participants is depicted in **Fig 1**. Twelve patients had no follow-up data. In total, 452 individuals with prediabetes completed the clinical follow-up, including the study cohort.

Baseline characteristics are summarized in **Table 1**. The mean age was 68.2±11.5 years, and 49.8% were male. The most common presentations were dyspnea (29.2%), atypical angina (27.2%), and typical angina (15.7%). Additionally, 54.2% had hypertension, 56.4% had dyslipidemia, 13.5% were smokers, and 12.2% had a history of heart failure. The mean LVEF within the entire study cohort was 61.7±17.6%.

**Table 1:** Baseline clinical and CMR characteristics of the study cohort.

	Total	Ischemia present	Ischemia absent	P-value
	(n = 452)	(n = 90)	(n = 362)	
Age (y)	68.2 ± 11.5	70.5 ± 10.5	67.7 ± 11.7	***0*.*03***
Male, n (%)	225 (49.8)	58 (64.4)	167 (46.1)	***0*.*02***
Body mass index (kg/m^2^)	25.5 ± 4.1	24.7 ± 3.7	25.6 ± 4.2	0.08
**Symptoms, n (%)**				
Chest pain	194 (42.9)	59 (65.5)	135 (37.3)	***<0*.*001***
• Typical angina	71 (15.7)	36 (40.0)	35 (9.97)	***<0*.*001***
• Atypical angina	123 (27.2)	23 (25.5)	100 (27.6)	0.79
Dyspnea	132 (29.2)	45 (50.0)	87 (24.0)	***<0*.*001***
**Medical history, n (%)**				
Hypertension	245 (54.2)	57 (63.3)	188 (51.9)	0.06
Hyperlipidemia	255 (56.4)	56 (62.2)	199 (55.0)	0.24
Smoking	61 (13.5)	20 (22.2)	41 (11.3)	***0*.*01***
Ischemic stroke	16 (3.5)	2 (2.2)	14 (3.9)	0.75
Family history of CAD	2 (0.4)	2 (2.2)	0 (0)	0.39
History of heart failure	55 (12.2)	15 (16.7)	40 (11.1)	0.15
**Laboratory data**				
Fasting plasma glucose (mg/dl)	110.8 ± 6.6	110.9 ± 6.6	110.7 ± 6.6	0.84
HbA1c (%)	6.0 ± 0.2	6.0 ± 0.2	6.0 ± 0.2	0.44
**Medications, n (%)**				
ACE inhibitor or ARB	126 (27.9)	28 (31.1)	98 (27.1)	0.43
Antiplatelet	182 (40.3)	49(54.4)	133 (36.7)	***0*.*03***
Beta-blocker	161 (35.6)	38 (42.2)	123 (34.0)	0.18
Calcium channel blocker	98 (21.7)	17 (18.9)	81 (22.4)	0.57
Statin	172 (38.1)	42 (46.7)	130 (36.0)	0.07
**CMR**				
LVEDV index (ml/m^2^)	87.3 ± 40.3	96.2 ± 44.6	85.1 ± 38.9	***0*.*02***
LVESV index (ml/m^2^)	39.2 ± 40.3	46.8 ± 45.8	37.3 ± 40.0	0.05
LV mass index (g/m^2^)	51.7 ± 18.6	59.0 ± 18.5	49.8 ± 18.2	***<0*.*001***
LV ejection fraction (%)	61.7 ± 17.6	58.2 ± 19.0	62.6 ± 17.0	***0*.*03***
Number of ischemic segments^a^	5 (3,7)	5 (3,7)	0 (0,0)	***<0*.*001***
LGE present, n (%)	121 (26.8)	55 (61.1)	66 (18.2)	***<0*.*001***
Number of LGE segments^b^	5 (2,8)	4.5 (2,6.5)	6 (2.5,8)	0.06

Values are n (%), mean ± SD, or median (25^th^, 75^th^ percentile). **Bold-italic** values are <0.05.

**Abbreviations:** ACE, angiotensin-converting enzyme; ARB, angiotensin II receptor blocker; CAD, coronary artery disease; CMR, cardiac magnetic resonance; HbA1c, hemoglobin A1c; LGE, late gadolinium enhancement; LV, left ventricular; LVEDV, left ventricular end-diastolic volume; LVESV, left ventricular end-systolic volume.

^a^ Only in patients with inducible ischemia.

^b^ Only in patients with LGE.

Inducible myocardial ischemia was presented in 90 patients, with an average of 5 ischemic segments (IQR 3, 7). One hundred and twenty-one patients exhibited LGE, all demonstrating a CAD pattern, either subendocardial or transmural LGE. Those with inducible ischemia, compared to those without, were older, more frequently male, presented more often with typical angina, and had a higher usage of antiplatelets (p<0.05 for all). Additionally, patients with inducible ischemia showed a higher LV mass index (59.0±18.5 vs. 49.8±18.2 ml/m^2^, p<0.001), lower LVEF (58.2±19.0% vs. 62.6±17.0%, p = 0.03), and a greater prevalence of LGE (61.1% vs. 18.2%, p<0.001).

### Patient outcomes

All patients within the cohort had available follow-up data, with a median follow-up duration of 8.1 years (IQR 5.7, 10.4 years). **S1 Table** presents cardiovascular events in the cohort, including 55 MACE (12.2%), 5 cardiovascular deaths (1.1%), 9 nonfatal MIs (2.0%), 12 hospitalizations for heart failure (2.6%), 9 ischemic strokes (2.0%), and 32 late coronary revascularizations (7.0%).

**Fig 2:**
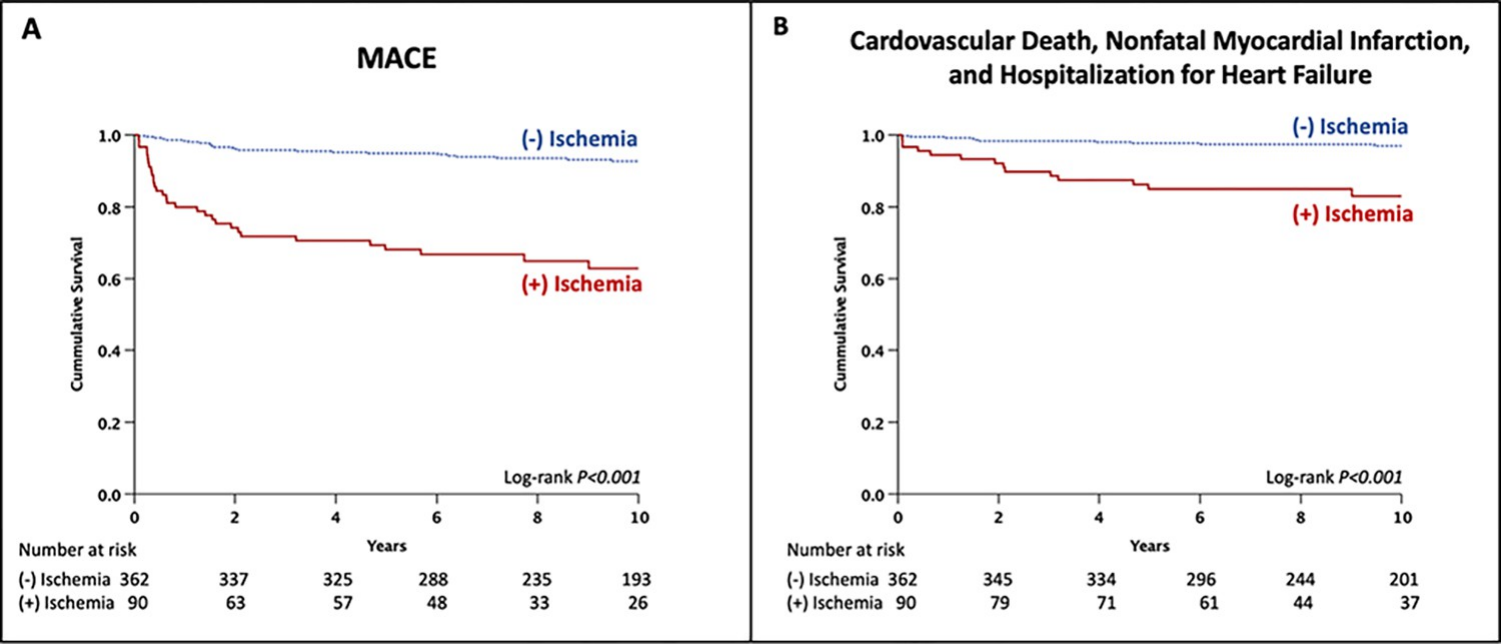
Kaplan–Meier survival curves for MACE (A) and the secondary endpoint (B) stratified by the presence of inducible ischemia. **Abbreviation:** MACE, major adverse cardiovascular events.

Kaplan–Meier analysis for survival free from MACE and the secondary endpoints, stratified by the presence or absence of inducible ischemia, is shown in **Fig 2.** Patients with inducible ischemia had significantly higher rates of MACE **(Fig 2A)** and the secondary endpoint **(Fig 2B)** (log-rank p<0.001 for both).

**Fig 3:**
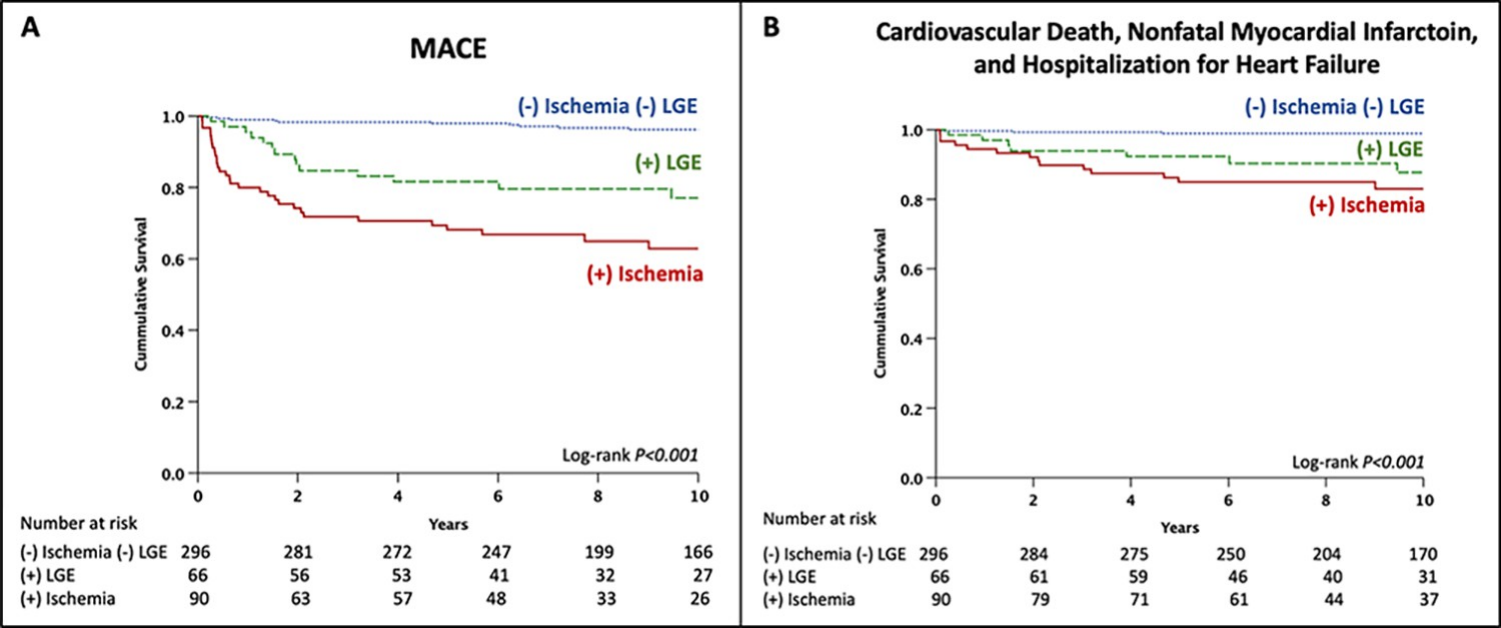
Kaplan–Meier survival curves for MACE (A) and the secondary endpoint (B) stratified by the presence of inducible ischemia and LGE. **Abbreviations:** LGE, late gadolinium enhancement; MACE, major adverse cardiovascular events.

**Fig 3** presents Kaplan–Meier analysis stratified by the presence of LGE (green line) or inducible ischemia (red line) for MACE **(Fig 3A)** and the secondary endpoint **(Fig 3B)**. It demonstrates significantly lower event-free survival for both patients with LGE and those with inducible ischemia (log-rank p<0.001 for both) compared to those without both LGE and inducible ischemia (blue dotted lines).

### Annual event rates

**Fig 4:**
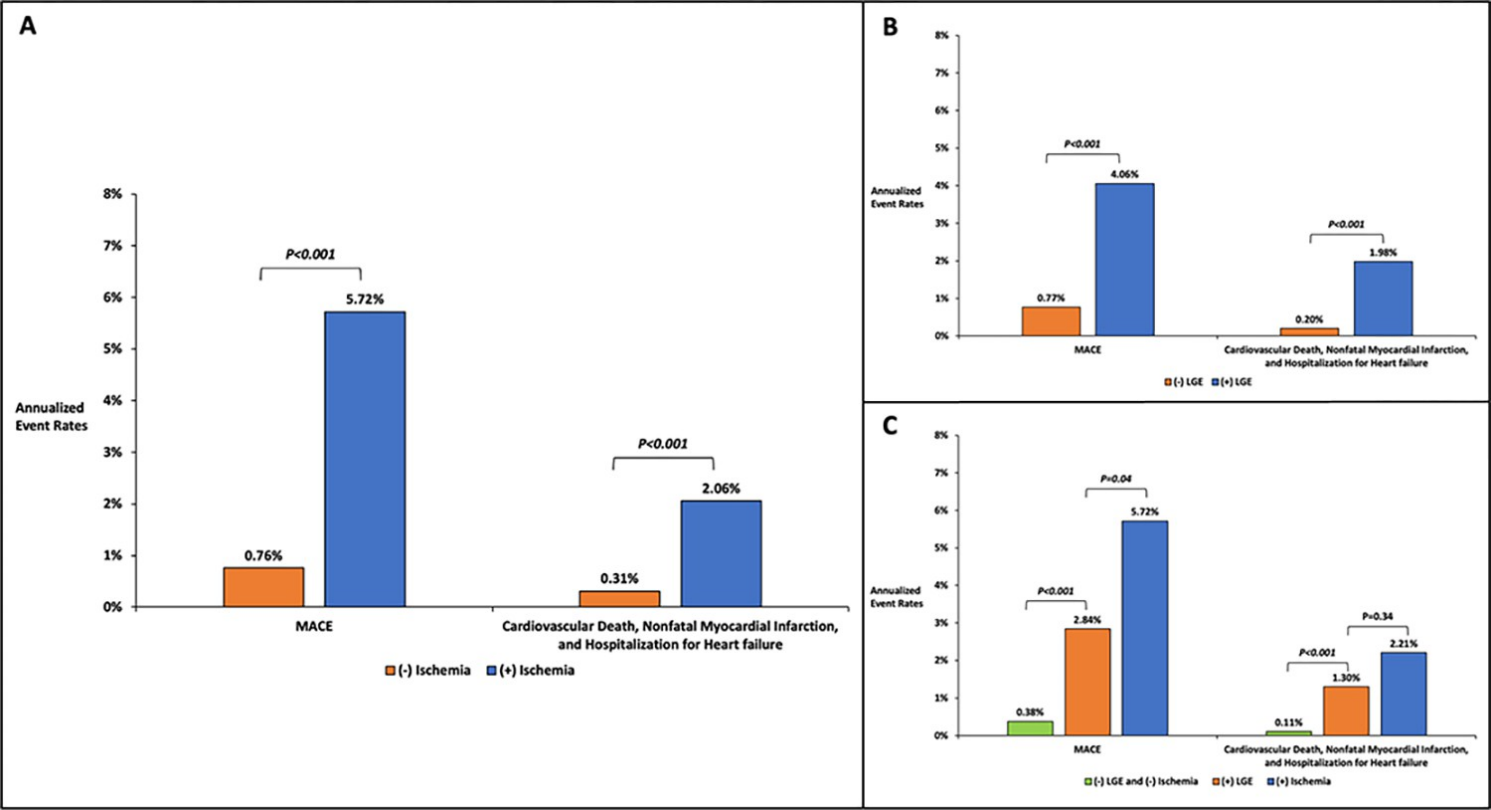
Annualized event rates for MACE and the secondary endpoint stratified by the presence of inducible ischemia (A), LGE (B), and both ischemia and LGE (C). **Abbreviations:** LGE, late gadolinium enhancement; MACE, major adverse cardiovascular events.

The entire cohort experienced an annual rate of MACE and the secondary endpoint of 1.50% (95% confidence interval [CI] 1.13%-1.94%) and 0.65% (95% CI 0.42%-0.97%), respectively. **Fig 4** displays the annualized event rates for MACE and the secondary endpoint, categorized based on the presence of inducible ischemia. Patients with inducible ischemia had significantly higher rates of MACE and the secondary endpoints than those without ischemia (5.72% vs. 0.76%, p<0.001 for MACE and 2.06% vs. 0.31%, p<0.001 for the secondary endpoint). Estimated annualized event rates for MACE and the secondary endpoint, stratified by the presence of LGE, are shown in **Fig 4A.** Patients with LGE had significantly higher rates of MACE and the secondary endpoints than those without LGE (4.06% vs. 0.77%, p<0.001 for MACE and 1.98% vs. 0.20%, p<0.001 for the secondary endpoint) **(Fig 4B)**.

**Fig 4C** illustrates the annualized event rates for MACE and the secondary endpoint across different patient groups: those without inducible ischemia or LGE (represented by green bars), those with LGE but no inducible ischemia (represented by orange bars), and those with inducible ischemia (represented by blue bars). Patients without ischemia or LGE had very low rates for MACE and the secondary endpoint (0.38% and 0.11%, respectively). For MACE and the secondary endpoint, patients with LGE had significantly higher annualized event rates than those without inducible ischemia or LGE (p<0.001 for both). Patients with inducible ischemia also had higher annualized event rates than patients with LGE only; however, this difference was not statistically significant for the secondary endpoint.

### Univariable and multivariable analyses of the primary and secondary endpoints

**Table 2** shows the results of the univariable Cox regression analysis for MACE. Body mass index (BMI), typical angina, dyspnea, history of heart failure, antiplatelet use, statin use, LV end-diastolic volume (EDV) index, LV end-systolic volume (ESV) index, LV mass index, LVEF, presence of inducible ischemia, the number of ischemic segments, and the presence of LGE were all identified as significant predictors of MACE. Multivariable Cox regression analysis was performed with stepwise selection of significant univariable predictors (p<0.05). The analyses revealed that both the presence of inducible ischemia (model 1) and the number of ischemic segments (model 2) were independent predictors for MACE (hazard ratio [HR] 3.36, 95% CI 1.90–5.94, p<0.001 for ischemia present and HR 1.14, 95% CI 1.06–1.22, p<0.001 for the number of ischemic segments). Other independent predictors included typical angina (HR 2.78, 95% CI 1.58–4.86, p<0.001), antiplatelet use (HR 2.69, 95% CI 1.49–4.85, p = 0.001), and LV mass index (HR 1.02, 95% CI 1.005–1.03, p = 0.006).

**Table 2:** Univariable and multivariable Cox regression analyses for the prediction of MACE.

	Univariable analysis		Multivariable analysis			
			Model 1^a^		Model 2^b^	
	Hazard ratio	P-value	Hazard ratio	P-value	Hazard ratio	P-value
	(95% CI)		(95% CI)		(95% CI)	
Age (y)	0.99 (0.97, 1.01)	0.48				
Male	1.71 (0.99, 2.93)	0.05				
Body mass index (kg/m^2^)	0.91 (0.85, 0.98)	***0*.*01***				
Typical angina	5.44 (3.21, 9.20)	***<0*.*001***	2.78 (1.58, 4.86)	***<0*.*001***	2.58 (1.44, 4.63)	***0*.*01***
Atypical angina	0.95 (0.52, 1.71)	0.86				
Dyspnea	3.17 (1.87, 5.35)	***<0*.*001***				
Hypertension	1.004 (0.59, 1.70)	0.99				
Hyperlipidemia	1.06 (0.63, 1.81)	0.81				
Smoking	1.28 (0.63, 2.62)	0.49				
Ischemic stroke	2.32 (0.84, 6.43)	0.10				
History of heart failure	2.88 (1.57, 5.29)	***0*.*001***				
Fasting plasma glucose (mg/dl)	1.03 (0.98, 1.08)	0.22				
HbA1c (%)	3.25 (0.25, 42.10)	0.36				
ACE inhibitor or ARB	1.17 (0.66, 2.07)	0.58				
Antiplatelet	3.78 (2.13, 6.67)	***<0*.*001***	2.69 (1.49, 4.85)	***0*.*001***	2.59 (1.41, 4.75)	***0*.*002***
Beta-blocker	1.65 (0.98, 2.79)	0.06				
Calcium channel blocker	0.58 (0.27, 1.23)	0.16				
Statin	2.35 (1.39, 3.99)	***0*.*002***				
LVEDV index (ml/m^2^)	1.01 (1.006, 1.014)	***<0*.*001***				
LVESV index (ml/m^2^)	1.01 (1.006, 1.013)	***<0*.*001***				
LV mass index (g/m^2^)	1.03 (1.02, 1.04)	***<0*.*001***	1.02 (1.005, 1.03)	***0*.*006***	1.02 (1.002, 1.03)	***0*.*02***
LV ejection fraction (%)	0.97 (0.96, 0.98)	***<0*.*001***				
Inducible ischemia present	6.25 (3.68, 10.60)	***<0*.*001***	3.36 (1.90, 5.94)	***<0*.*001***		
Number of ischemic segments	1.23 (1.16, 1.30)	***<0*.*001***			1.14 (1.06, 1.22)	***<0*.*001***
LGE present	4.67 (2.73, 8.06)	***<0*.*001***				

**Bold-italic** values are <0.05.

**Abbreviations:** ACE, angiotensin-converting enzyme; ARB, angiotensin II receptor blocker; CAD, coronary artery disease; CI, confidence interval; CMR, cardiac magnetic resonance; HbA1c, hemoglobin A1c; LGE, late gadolinium enhancement; LV, left ventricular; LVEDV, left ventricular end-diastolic volume; LVESV, left ventricular end-systolic volume; MACE, major adverse cardiovascular events.

^a^ Inducible ischemia was included as a categorical variable (presence or absence).

^b^ Inducible ischemia was included as a continuous variable (per-segment extent).

**Table 3** presents the results of the univariable Cox regression analysis for the secondary endpoint, comprising cardiovascular death, nonfatal MI, or hospitalization for heart failure. BMI, typical angina, dyspnea, history of heart failure, antiplatelet use, statin use, LVEDV index, LVESV index, LV mass index, LVEF, presence of inducible ischemia, the number of ischemic segments, and the presence of LGE were all identified as significant predictors of MACE. Multivariable Cox regression analysis revealed that the presence of inducible ischemia was an independent predictor for the secondary endpoint (HR 2.89, 95% CI 1.22–6.80, p = 0.01). Other independent predictors included typical angina (HR 2.74, 95% CI 1.18–6.39, p = 0.02), history of heart failure (HR 2.96, 95%CI 1.27–6.88, p = 0.01), LV mass index (HR 1.02, 95%CI 1.004–1.04, p = 0.01), and the presence of LGE (HR 3.56, 95%CI 1.25–10.13, p = 0.02).

**Table 3:** Univariable and multivariable Cox regression analyses for the prediction of the secondary endpoint.

	Univariable analysis		Multivariable analysis			
			Model 1^a^		Model 2^b^	
	Hazard ratio	P-value	Hazard ratio	P-value	Hazard ratio	P-value
	(95% CI)		(95% CI)		(95% CI)	
Age (y)	0.99 (0.96, 1.03)	0.78				
Male	1.67 (0.73, 3.83)	0.22				
Body mass index (kg/m^2^)	0.86 (0.77, 0.96)	***0*.*009***				
Typical angina	5.64 (2.53, 12.55)	***<0*.*001***	2.74 (1.18, 6.39)	***0*.*02***	3.23 (1.38, 7.58)	***0*.*007***
Atypical angina	0.51 (0.17, 1.48)	0.21				
Dyspnea	4.28 (1.87, 9.78)	***0*.*001***				
Hypertension	0.61 (0.27, 1.37)	0.23				
Hyperlipidemia	0.67 (0.30, 1.50)	0.33				
Smoking	0.92 (0.27, 3.10)	0.90				
Ischemic stroke	1.21 (0.16, 9.01)	0.85				
History of heart failure	6.29 (2.78, 14.23)	***<0*.*001***	2.96 (1.27, 6.88)	***0*.*01***	2.51 (1.07, 5.88)	***0*.*03***
Fasting plasma glucose (mg/dl)	1.03 (0.96, 1.09)	0.37				
HbA1c (%)	9.03 (0.15, 523.64)	0.29				
ACE inhibitor or ARB	1.11 (0.46, 2.68)	0.81				
Antiplatelet	5.94 (2.22, 15.91)	***<0*.*001***				
Beta-blocker	1.85 (0.83, 4.11)	0.13				
Calcium channel blocker	0.95 (0.35, 2.54)	0.92				
Statin	2.40 (1.07, 5.42)	***0*.*03***				
LVEDV index (ml/m^2^)	1.01 (1.009, 1.02)	***<0*.*001***				
LVESV index (ml/m^2^)	1.01 (1.009, 1.02)	***<0*.*001***				
LV mass index (g/m^2^)	1.03 (1.02, 1.04)	***<0*.*001***	1.02 (1.004, 1.04)	***0*.*01***	1.02 (1.004, 1.04)	***0*.*02***
LV ejection fraction (%)	0.96 (0.94, 0.97)	***<0*.*001***				
Inducible ischemia present	6.17 (2.74, 13.90)	***<0*.*001***	2.89 (1.22, 6.80)	***0*.*01***		
Number of ischemic segments	1.20 (1.09, 1.31)	***<0*.*001***				
LGE present	9.04 (3.58, 22.79)	***<0*.*001***			3.56 (1.25, 10.13)	***0*.*02***

**Bold-italic** values are < 0.05.

**Abbreviations:** ACE, angiotensin-converting enzyme; ARB, angiotensin II receptor blocker; CAD, coronary artery disease; CI, confidence interval; CMR, cardiac magnetic resonance; LGE, late gadolinium enhancement; LV, left ventricular; LVEDV, left ventricular end-diastolic volume; LVESV, left ventricular end-systolic volume.

^a^ Inducible ischemia was included as a categorical variable (presence or absence).

^b^ Inducible ischemia was included as a continuous variable (per-segment extent).

### Incremental prognostic value of stress perfusion CMR

**Table 4** illustrates the discriminatory power and reclassification associated with inducible ischemia and LGE in predicting MACE. The baseline model, utilizing stepwise variable selection, yielded C-statistic values of 0.77 (95% CI 0.72–0.83). The inclusion of inducible ischemia notably enhanced the C-statistic to 0.82 (95% CI 0.76–0.88; NRI = 0.43, p = 0.02; IDI = 0.04, p = 0.04). Moreover, the incorporation of both inducible ischemia and LGE further elevated the C-statistic to 0.83 (95% CI 0.78–0.87; NRI = 0.42, p<0.001; IDI = 0.05, p<0.001).

**Table 4:** Discrimination and reclassification associated with inducible ischemia and LGE for prediction of MACE.

	C-index (95% CI)	NRI (95% CI)	P-value	IDI (95% CI)	P-value
Baseline model^a^	0.77 (0.72, 0.83)	Reference	Reference	Reference	Reference
Baseline model^a^ + ischemia	0.82 (0.76, 0.88)	0.43 (0.20, 0.55)	***0*.*02***	0.04 (0.00, 0.11)	***0*.*04***
Baseline model^a^ + ischemia + LGE	0.83 (0.78, 0.87)	0.42 (0.30, 0.57)	***<0*.*001***	0.05 (0.01, 0.13)	***<0*.*001***

**Bold-italic** values are <0.05.

^a^ Baseline model includes typical angina, antiplatelet use, and LV mass index.

**Abbreviations:** CI, confidence interval; IDI, integrated discrimination index; LGE, late gadolinium enhancement; LV, left ventricular; MACE, major adverse cardiovascular events; NRI, net reclassification index.

## Discussion

The findings of the study can be summarized as follows: 1) Among patients with prediabetes and suspected CAD referred for adenosine stress perfusion CMR, the presence and extent of inducible ischemia emerged as robust and independent predictors of MACE and the secondary endpoint. 2) LGE independently predicted the secondary endpoint. 3) The inclusion of inducible ischemia and LGE improved the discrimination and reclassification of the model in predicting MACE, even after adjusting for clinical factors. 4) Patients without inducible ischemia or LGE on CMR exhibited notably lower annualized event rates for both MACEs (0.76%) and the secondary endpoint (0.31%).

Prediabetes not only augments the risk of developing diabetes but also enhances susceptibility to cardiovascular disease, presenting a 20% elevated likelihood compared to normoglycemic individuals, particularly with respect to CAD [20]. Stress perfusion CMR is one of the preferred modalities to detect and characterize CAD. In our study, inducible ischemia was detected in 19% of prediabetic patients. The prevalence of inducible ischemia in our study was relatively low compared to previous studies [21, 22]. This could be attributed to our study’s inclusion criteria, which focused solely on patients with suspected CAD. Those with inducible ischemia were older, predominantly male, presented with typical angina, had a higher prevalence of smoking, and were more likely to use antiplatelets than those without ischemia. Patients with inducible ischemia exhibited higher LV volumes, a higher LV mass index, and lower LVEF.

Unlike prediabetes, functional stress testing has been extensively studied in patients with diabetes mellitus who have known or suspected CAD [12, 23, 24]. This encompasses stress perfusion CMR [12]. Heydari et al. investigated the prognostic significance of stress perfusion CMR in 173 symptomatic diabetic individuals, noting that those without inducible ischemia had an annualized event rate of 1.4%, contrasting with 8.2% (p = 0.0003) in individuals with inducible ischemia [12]. The presence of inducible ischemia additionally accurately reassigned over 40% of the current diabetic cohort into clinically relevant risk categories [12]. Our study is the first to demonstrate the prognostic value of stress perfusion CMR in patients with prediabetes and suspected CAD. In our study, prediabetic patients with inducible ischemia exhibited significantly higher rates of MACE compared to patients without ischemia, with an annualized event rate of 5.72% vs. 0.76%, p < 0.001. Additionally, for the secondary endpoint, which includes a composite of cardiovascular death, nonfatal MI, and hospitalization for heart failure, the rates were 2.06% vs. 0.31%, p<0.001. Multivariable analysis also showed that inducible ischemia is the strongest independent predictor of MACE. These findings align with previous stress perfusion CMR studies that have demonstrated the presence of inducible ischemia as an important predictor of clinical outcomes [25–28].

Instead of solely the presence of ischemia, an ischemic burden was also identified as a significant predictor of MACE. Vincenti et al. showed that an ischemic burden of ≥1.5 segments was the most robust predictor in individuals with known or suspected CAD who underwent stress perfusion CMR [22]. Our results similarly revealed that the number of ischemic segments was an independent predictor of MACE, with a hazard ratio of 1.14 per ischemic segment.

CMR not only detects inducible ischemia but also offers a unique advantage in identifying myocardial infarctions using LGE imaging, even in patients with normal LV function [29]. Subendocardial or transmural LGE in patients without a history of MI, known as ’unrecognized MI (UMI),’ has been reported in several CMR studies. For instance, a multicenter study by Antiochos et al. found a UMI prevalence of 14.8% in patients referred for stress CMR to assess CAD [30]. UMI is relatively common in patients with diabetes mellitus. Kwong et al. demonstrated that 28% of diabetic patients clinically referred for CMR had UMI [31], while Elliott et al. similarly found that 28% of asymptomatic high-risk diabetic patients had UMI [32]. UMI was strongly associated with cardiac events, including mortality, in both studies [31, 32]. Since our study included patients with prediabetes without a history of CAD, the prevalence of UMI was 26.8%, which was higher compared to the study by Antiochos et al. [30] However, our prevalence was closer to that observed in patients with diabetes mellitus in the studies by Kwong et al. [31] and Elliott et al. [32]. Several factors could explain the relatively high prevalence of UMI in our study: (1) our patients were older than those in other studies, (2) they had a higher prevalence of heart failure, and (3) all of our patients were clinically referred for CMR to assess CAD, meaning they typically had a higher risk profile and pretest probability of obstructive CAD or MI, as lower-risk patients in a developing country like Thailand are generally referred for exercise stress tests or stress echocardiography. Nevertheless, in our study, prediabetic patients with LGE (indicating UMI) had a higher rate of MACE and secondary endpoints than those without LGE. LGE was also the strongest predictor of cardiac death, nonfatal MI, and hospitalization for heart failure. Overall, UMI in our study was independently associated with a worse prognosis, consistent with previous findings [30–32].

Inducible ischemia provided incremental prognostic value over clinical factors and LV mass index for predicting MACE. The addition of LGE to ischemia significantly improved the model’s discrimination for MACE compared to ischemia alone. The combination of ischemia and LGE has been shown to have prognostic value beyond ischemia or LGE alone in previous studies [21, 33]. Our data also confirm the incremental prognostic value of ischemia and LGE in patients with prediabetes. This is a unique advantage of stress perfusion CMR, providing a comprehensive assessment of LV function, ischemia, and LGE in a single study. Overall, our data support the use of stress perfusion CMR to evaluate patients with prediabetes and suspected CAD.

Our study had several limitations. First, it was a retrospective study that only included prediabetic patients referred for stress perfusion CMR, which introduces a potential selection bias and may limit the generalizability of the results to the broader population of prediabetic patients. Second, a total of 12 patients (2.6%) were lost to follow-up, possibly due to the relatively long follow-up period. Third, we included only revascularization procedures that occurred 90 days after the CMR procedures, as we chose not to incorporate revascularization procedures that could be influenced by CMR results. This approach might have underestimated the number of events in our results. However, when we analyzed the secondary endpoint, excluding revascularization procedures, inducible ischemia, and LGE still emerged as independent predictors of adverse outcomes. Fourth, we did not conduct a separate analysis to assess the prognostic value of CMR in patients with IFG and raised HbA1c due to limitations in the population and event rate. However, it’s worth noting that both IFG and raised HbA1c have been associated with an increased risk of cardiovascular events [34].

## Conclusion

In patients with prediabetes and suspected CAD, inducible myocardial ischemia and LGE using CMR demonstrate important prognostic value for predicting cardiovascular death, nonfatal MI, hospitalization for heart failure, ischemic stroke, and coronary revascularization over traditional risk factors. Stress perfusion CMR also offer independent prognostic utility and effectively reclassify risk in patients with prediabetes and suspected CAD.

## Supporting information

S1 Table Patient outcomes during the follow-up period. (DOCX)

## Abbreviations

CAD: coronary artery disease
CI: confidence interval
CMR: cardiac magnetic resonance
EDV: end-diastolic volume
EF: ejection fraction
ESV: end-systolic volume
HR: hazard ratio
IDI: integrative discrimination index
LV: left ventricle
LGE: late gadolinium enhancement
LVEF: left ventricular ejection fraction
MACE: major adverse cardiovascular events
MI: myocardial infarction
NRI: net reclassification improvement
UMI: unrecognized myocardial infarction
