# Supplementary material for: Prognostic value of stress perfusion cardiac magnetic resonance in patients with prediabetes and suspected coronary artery disease
Source: PLoS One. 2024 Oct 10;19(10):e0311875. doi: 10.1371/journal.pone.0311875 (PMC11466432; doi:10.1371/journal.pone.0311875)
Supplement: S1 Table — (DOCX) [file pone.0311875.s001.docx]

**S1 Table.** Patient outcomes during the follow-up period.

|  | Total | Ischemia present | Ischemia absent |
| --- | --- | --- | --- |
|  | (n=452) | (n=90) | (n=362) |
| MACE | 55 (12.2) | 31 (34.4) | 25 (6.9) |
| Cardiovascular death | 5 (1.1) | 2 (2.2) | 3 (0.8) |
| Nonfatal myocardial infarction | 9 (2.0) | 9 (10.0) | 0 (0) |
| Hospitalization for heart failure | 12 (2.6) | 5 (5.6) | 7 (1.9) |
| Ischemic stroke | 9 (2.0) | 4 (4.4) | 5 (1.4) |
| Late coronary revascularization | 32 (7.0) | 23 (25.6) | 9 (2.5) |

Values are n (%).

MACE was defined as a composite of cardiovascular death, nonfatal MI, hospitalization for heart failure, ischemic stroke, and late coronary revascularization (>90 days post-CMR).

**Abbreviation:** MACE, major adverse cardiovascular events.
